# Digital tools in the informed consent process: a systematic review

**DOI:** 10.1186/s12910-021-00585-8

**Published:** 2021-02-27

**Authors:** Francesco Gesualdo, Margherita Daverio, Laura Palazzani, Dimitris Dimitriou, Javier Diez-Domingo, Jaime Fons-Martinez, Sally Jackson, Pascal Vignally, Caterina Rizzo, Alberto Eugenio Tozzi

**Affiliations:** 1 Ospedale Pediatrico Bambino Gesù (OPBG), Piazza di Sant’Onofrio, 4, 00165 Rome, Italy; 2 Libera Università Maria Ss. Assunta (LUMSA), Via della Traspontina, 21, 00193 Rome, Italy; 3 AND Consulting Group SPRL, Place Marcel Broodthaers, 8, 1060 Brussels, Belgium; 4 The Foundation for the Promotion of Health and Biomedical Research of Valencia Region (FISABIO), Avda. de Catalunya, 21, 46020 Valencia, Spain

**Keywords:** RCT, Informed consent, Innovation, Multimedia, Video, Digital tools, Systematic review

## Abstract

**Background:**

Providing understandable information to patients is necessary to achieve the aims of the Informed Consent process: respecting and promoting patients’ autonomy and protecting patients from harm. In recent decades, new, primarily digital technologies have been used to apply and test innovative formats of Informed Consent. We conducted a systematic review to explore the impact of using digital tools for Informed Consent in both clinical research and in clinical practice. Understanding, satisfaction and participation were compared for digital tools versus the non-digital Informed Consent process.

**Methods:**

We searched for studies on available electronic databases, including Pubmed, EMBASE, and Cochrane. Studies were identified using specific Mesh-terms/keywords. We included studies, published from January 2012 to October 2020, that focused on the use of digital Informed Consent tools for clinical research, or clinical procedures. Digital interventions were defined as interventions that used multimedia or audio–video to provide information to patients. We classified the interventions into 3 different categories: video only, non-interactive multimedia, and interactive multimedia.

**Results:**

Our search yielded 19,579 publications. After title and abstract screening 100 studies were retained for full-text analysis, of which 73 publications were included. Studies examined interactive multimedia (29/73), non-interactive multimedia (13/73), and videos (31/73), and most (34/38) studies were conducted on adults. Innovations in consent were tested for clinical/surgical procedures (26/38) and clinical research (12/38). For research IC, 21 outcomes were explored, with a positive effect on at least one of the studied outcomes being observed in 8/12 studies. For clinical/surgical procedures 49 outcomes were explored, and 21/26 studies reported a positive effect on at least one of the studied outcomes.

**Conclusions:**

Digital technologies for informed consent were not found to negatively affect any of the outcomes, and overall, multimedia tools seem desirable. Multimedia tools indicated a higher impact than videos only. Presence of a researcher may potentially enhance efficacy of different outcomes in research IC processes. Studies were heterogeneous in design, making evaluation of impact challenging. Robust study design including standardization is needed to conclusively assess impact.

## Background

In 1967, the World Medical Association Declaration of Helsinki [1] set the framework for the practical application of the notion of Informed Consent (IC) in clinical research for the years to come. The declaration built upon the foundations put in place by the Nuremberg Code, which stated that the primary consideration in research is the subject’s voluntary consent [2]. After more than a half a century, these principles are still valid.

In clinical research, the IC process is essential for the potential participant to be informed of the fundamental elements of the research protocol, of the possible benefits but also of the risks and of the level of uncertainty relating to the research project, in order to be able to choose freely and consciously [1]. Ethical [3] and legal [4] requirements are clear in recommending and regulating an adequate IC process as a key element of clinical research. In the disclosure of the information, therapeutic misconception [5] or unrealistic optimism of the participant should be taken into account, as they are factors that can prevent the subject from understanding correctly the risks that a clinical study can imply. This can happen because of an overestimation of envisaged benefits deriving from participating in a clinical trial [6] and/or due to misunderstandings concerning clinical research procedures (e.g. about randomization and/or the role of placebos in clinical trials) [7].

On the clinical practice side, providing understandable information to patients is also necessary, in order to achieve the two important aims of respecting and promoting patients’ autonomy and protecting patients from harm [8]. In the health care context, the specific function of the IC is to provide an instrument to guarantee a balanced physician–patient relationship: it is an explicit expression and authorization given by the patient to accept (consent) or refuse (dissent) treatments or clinical/surgical procedures offered by the doctor [9]. An intervention in the health field may only be carried out after the patient has given free and informed consent to it [10]. Both in clinical practice and in clinical research, a clear and complete information process, which includes the disclosure of information and its comprehension [11], is the condition for providing a valid consent [12].

Research participants’ and patients’ comprehension of IC is therefore crucial. Nevertheless, frequently, comprehension can be too limited for an autonomous decision to be made. A meta-analysis conducted on 135 cohorts of participants in clinical trials showed that IC comprehension varied between 52 and 76% for different components [13] and only one third of study participants in pre-surgery studies published before 2006 showed a correct understanding of risks associated with surgery [14]. According to Tam et al., the proportion of participants understanding IC documents has not increased over the past 30 years [13].

IC comprehension can be affected by a number of factors that should be taken into account in designing an adequate IC process.

First, age, gender, and health literacy may affect the communication process and the comprehension of the IC, and therefore bias the decisions taken by patients [15–18]; differences in cultural background among the researcher/physician and the participant/patient can have an influence on the information process [19], and comprehension of the disclosed information can vary in high and low income countries [20].

Secondly, context-dependent factors (e.g. clinical and affective factors) may come into play, for example depending on the clinical conditions of the participant/patient, as in the case of phase I trials, where patients normally do not have another alternative to treatment [21, 22]. Moreover, trust can support the IC process [23] but it cannot overcome the role of the information provided [24]. If trust outweighs information, it may generate the so-called researcher bias [25].

Thirdly, comprehension of IC can be hampered by elements directly related to the format of the information provided to participants. The format affects the readability of consent documents, which is often insufficient [26], due to complex contents and the length of the text.

In this perspective, digital tools can be adopted in IC processes with different potential impacts: improving comprehension of the disclosed information, addressing IC-related issues (e.g. therapeutic misconception, researcher bias) by improving the information process, and improving an informed participation of vulnerable populations in clinical research (e.g. minors, subjects coming from different cultural and religious backgrounds, persons with disabilities) through tailored communication [27]. To facilitate an informed decision, effective techniques are required to communicate abstract concepts such as experimental study methods, and enable their comprehension, as in many cases patients may decide to participate in a study or express satisfaction towards a consent format without having a comprehensive understanding of its contents [28].

Several studies have aimed to improve the access and comprehension of the IC format, by providing information using a diverse range of digital instruments including videos, audio–video formats, and computer-based techniques [29, 30]. Previous published meta-analyses have shown a limited effect of multimedia in improving understanding during the IC process in clinical research [28, 31]; they also reported that interventions on IC through digital or multimedia tools do not negatively affect patients’ satisfaction [28, 31]. Several different outcome measures have been taken into account throughout different studies, but results are often inconsistent, and the generalisability of studies is limited; the review by Nishimura reported the need for the identification of best practices of IC interventions for next systematic comparisons [31]. At present, no evidence of the impact of specific, digitally-supported IC processes is available.

We conducted a systematic review to assess the impact of digitally-supported IC processes on understanding, satisfaction, anxiety and participation compared with non-digital IC processes, in the context of a H2020 funded project dedicated to improving the IC process in biomedical/clinical research (i-CONSENT, Grant Agreement No. 741856). We took into account studies reporting the information process both in clinical research and in clinical/surgical procedures, in consideration of the key role that a correct and understandable information plays in the consent process in both settings (clinical research and healthcare contexts).

## Methods

Our study was conducted following the Preferred Reporting Items for Systematic reviews and Meta-Analyses (PRISMA) guidelines [32].

### Search strategy

We conducted a systematic literature review following an a-priori defined, unpublished protocol. We searched for studies published between 1st January 2012 and 31st October 2020 on available electronic databases including Pubmed, Embase and Cochrane. The term “Informed Consent” and related terms were combined with keywords or Mesh terms related to technologies considered relevant for innovative, digitally supported IC processes (see Additional file 1 for details). The reference list of published reviews were screened for relevant articles meeting the eligibility criteria.

### Eligibility criteria

We included studies published from January 2012 to October 2020, with full text available in English, Italian or French, which compared the effect of digital IC vs. non-digital forms of IC (written on paper and/or face-to-face discussion) for participation in research studies or for clinical procedures. Digital interventions were defined as interventions that used multimedia or audio-visual means to provide information to patients. We selected studies focusing on digital tools both for clinical IC (for surgery, diagnostic procedures, therapeutic interventions) and for research IC. Results will be presented in two different sections for these two types of consent.

In order to review more informative and robust studies providing information on the existing differences between digital and non-digital IC processes, we decided to select only articles based on a randomised controlled trial (RCT) study design. Therefore, we excluded articles that reported the results of cohort studies, systematic reviews or meta-analyses.

### Study selection

One researcher (PV) screened the titles and abstracts of the unique references to identify potentially relevant papers. After this primary screening, full texts were reviewed to assess eligibility criteria for inclusion in the review.

### Data extraction and definitions

Data were extracted by two researchers (CR and PV), using a standardized extraction form. The two datasets were then evaluated and in case of conflicting results a decision was taken through a discussion between CR, PV and a third researcher (AET).

For each study, we extracted the following information: population and setting; type of IC intervention (video, interactive multimedia, non-interactive multimedia); kind of non-digital IC process used in the comparison group; type of study/procedure for which the consent was requested (clinical study, diagnostic test, therapy/vaccine, surgery); outcome measured (knowledge/comprehension/understanding/recall, satisfaction, acceptability, anxiety, study participation) and effect value for the comparison of the intervention and control groups.

For each article, we also reported if the article addressed the concepts of therapeutic misconception or of researcher/clinician allegiance in the recruitment process.

Quality of included studies was assessed using criteria selected through discussion among the involved researchers: sufficient sample size (according to a priori or post-hoc sample size calculation—studies not reporting a sample size calculation were considered as not meeting the criteria); sufficient description (based on researchers’ judgement) of RCT or clinical procedure for which the consent was requested, intervention (digital tools in the consent process) and comparison; objective criteria to measure outcome; consideration of limitations (any limitation that affected both study arms equally, e.g. sample size); and consideration of bias (any element producing a differential effect on the two study arms).

Interventions were classified into 3 different categories: video only, non-interactive multimedia, and interactive multimedia. Video was defined as the provision of audio-visual content only. Multimedia interventions were defined as software that provided consent information in various format combinations (images, audio, videos, graphics, etc.). Multimedia interventions were either navigated directly by the patients or used by the researcher as a support during the explanation of the study/procedure. Interaction was defined as patient interaction with the software, eg. providing responses to questions. The non-digital format of the IC process was defined as reading a paper text presenting the IC and/or a standardized face-to-face discussion.

Regarding outcomes, the reported participation in the clinical study was either an actual participation, when the patient actually signed the IC for participating in the RCT or clinical procedures, or a hypothetical participation where patients declared their potential participation in a future RCT or clinical care procedures. Participant understanding of the IC document was a key outcome that we looked for. Studies meeting our eligibility criteria either referred to “understanding”, “comprehension”, “knowledge” or “recall”. As only a few of the included studies drew a distinction between these terms, in this review paper, we use the term “understanding” to refer to outcomes that may also have been termed “knowledge” and “comprehension”. Information retention and information recall were also categorised as understanding.

We classified an intervention as effective on a specific outcome if the article reported a statistically significant effect (irrespective of the effect magnitude) of the studied intervention with respect to the comparison.

### Data synthesis

Some of the retrieved data were categorised (kind of study/procedure for which the consent was requested, type of digital intervention, kind of outcome), and descriptive statistics were used to analyse the kind of interventions and main outcomes considered. We present a narrative synthesis of the main results. The positive effect of digital tools on each outcome was presented as the proportion of studies reporting statistically significant results (irrespective of the effect magnitude) on the total studies focusing on that specific outcome. Neutral effect of the digital intervention compared to non-digital IC process was considered as negative.

## Results

### Results of the literature search

**Fig. 1:**
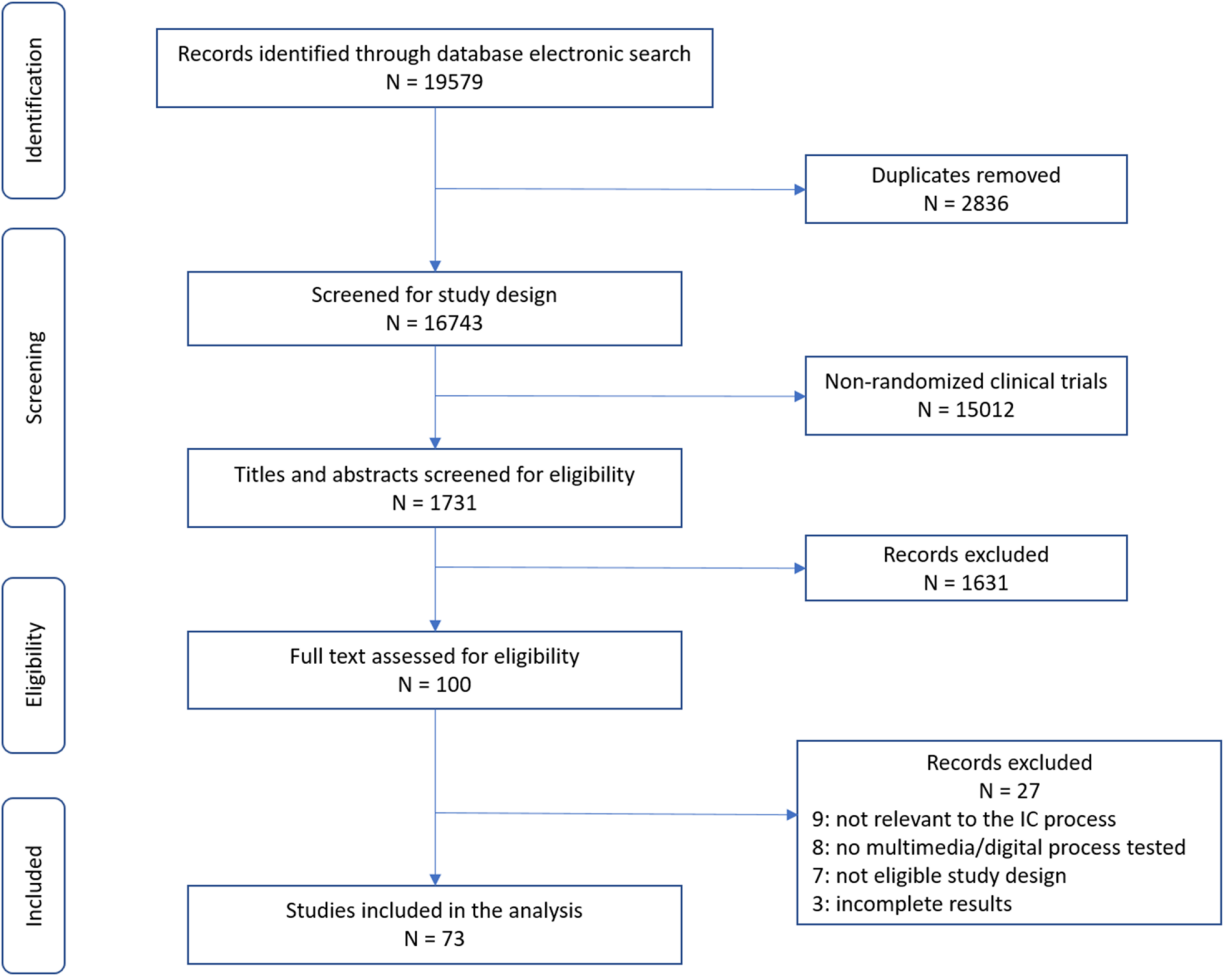
Flow diagram of the search process

We identified 19,579 publications through electronic search. A total of 16,743 were electronically screened to select clinical trials; after removing duplicates, 1,731 publications were screened for eligibility through reading title and abstract, 100 articles were retained for full text assessment and 73 were included in the review. Details of the study selection process are reported in Fig. 1. Studies included in the review are reported in Additional file 2 and 3.

The majority of the study populations included in the systematic review were adult individuals; 6.8% (5/73) of the studies investigated consent provided for children, and 2.7% (2/73)investigated assent by adolescents. Of the selected studies, 54.8% (40/73) were set in North America, 23.3% (17/73) in Europe, 9.6% (7/73) in Oceania, 9.6% (7/73) in Asia and 2.7% (2/73) in Africa.

Twenty-eight studies (38.4%) investigated digitally supported IC processes for research (see Additional file 2) and 45 studies (61.6%) investigated digitally supported consent processes for clinical/surgical procedures (see Additional file 3).

Overall, 29 studies used interactive multimedia (39.7%), 13 used non-interactive multimedia (17.8%), and 31 used videos (42.5%). Studied outcomes differed among included articles. Thirty-five (48%) articles explored more than one outcome.

With regards to the quality of the included studies, 46 (63.0%) had a sufficient sample size, justified by a power calculation; 70 (95.9%) reported an adequate description of the research/clinical procedure for which the consent was requested, 73 (100%) reported a sufficient description of the intervention and 71 (97.3%) reported a sufficient description of the comparison; 70 (95.9%) used objective criteria to measure the outcome; the researcher responsible for collecting information about the outcome was blinded to group allocation in 21 (29%) of the studies; 54 (74%) considered limitations and 33 (45.2%) considered bias.

### Research studies

A total of 28 studies reported results on the efficacy of digitally supported IC processes for research studies (Table 1). Among those, 16 were mock studies. Fifteen (53.6%) used interactive multimedia, 4 (14.3%) used non-interactive multimedia, and 9 (32.1%) used videos. Each of the included articles explored from 1 to 3 outcomes: 24 explored the effect of the digital intervention on understanding, 8 articles explored the effect on satisfaction and 10 on participation in a research study (which was hypothetical in 5 studies). None of the included studies investigated the effect of digitally-supported research IC on anxiety. Among the 28 included articles, 15 (53.6%) reported a positive effect on at least one of the studied outcomes. TMore than a half of studies investigating interactive multimedia interventions reported a positive effect on at least one of the studied outcomes: 8/15 (53.3%) for interactive multimedia interventions and 3/4 for non-interactive multimedia interventions. The proportion of studies reporting a positive effect was slightly lower for videos (4/9). A positive effect was reported in 12 (50.0%) of the 24 studies investigating understanding and in 5 of the 8 studies investigating satisfaction. On the other hand, participation in research studies was improved in 4/10 studies only and 3 out of these 4 were mock studies.

**Table 1:** Description of the positive effect of each investigated outcome (articles explored more than one outcome) by type of digital intervention and study

	N of studies	Understanding		Participation in the study/consent to clinical procedure		Satisfaction		Anxiety		At least one positive outcome	
		Positive/total	Percent positive	Positive/total	Percent positive	Positive/total	Percent positive	Positive/total	Percent positive	Positive/total	Percent positive
Research studies											
Video	9	2/7	28.6	1/4	25.0	1/1	100.0	–	–	4/9	44.4
Other multimedia non interactive	4	2/3	66.7	1/1	100.0	–	–	–	–	3/4	75.0
Multimedia interactive	15	8/14	57.1	2/5	44.0	4/7	57.1	–	–	8/15	53.3
Total	28	12/24	50.0	4/10	40.0	5/8	62.5	–	–	15/28	53.6
Clinical procedures											
Video	22	13/18	72.2	1/2	50.0	8/14	57.1	2/7	28.6	16/22	72.7
Other multimedia non interactive	9	5/7	71.4	1/1	100.0	3/6	50.0	2/3	66.7	8/9	88.9
Multimedia interactive	14	10/12	83.3	2/3	66.7	4/5	80.0	0/3	0.0	13/14	92.9
Total	45	28/37	75.7	4/6	66.7	15/25	60.0	4/13	30.8	37/45	82.2
All studies											
Video	31	15/25	60.0	2/6	33.3	9/15	60.0	2/7	28.6	20/31	64.5
Other multimedia non interactive	13	7/10	70.0	2/2	100.0	3/6	50.0	2/3	66.7	11/13	84.6
Multimedia interactive	29	18/26	69.2	4/8	50.0	8/12	66.7	0/3	0.0	21/29	72.4
Total	73	40/61	65.6	8/16	50.0	20/33	60.6	4/13	30.8	52/73	71.2

In 14 studies (50%), the researcher was present during the consent process and 9 (64.3%) had at least one positive outcome. On the other hand, among the 14 studies in which a researcher was not present during the IC process, 6 (42.9%) had at least one positive outcome.

### Clinical/surgical procedures

A total of 45 studies reported results on the efficacy of digitally supported IC processes for clinical/surgical procedures (Table 1). Of these, 5 were mock studies. The processes studied in the included articles were aimed at obtaining IC for surgery (86.7%), diagnostic tests (6.7%), and therapy/vaccine (6.7%).

Among these, 14 (31.1%) used interactive multimedia, 9 (20.0%) used non-interactive multimedia, and 22 (48.9%) used videos. Each of the included articles explored from 1 to 4 outcomes: 37 articles explored the effect of the digital intervention on understanding, 25 on satisfaction, 13 on anxiety, and 6 on participation. Of these, 4 were mock studies. The IC under evaluation was developed with the active participation of patients in 2 (4%) of the studies dedicated to clinical IC. Two articles addressed the concept of therapeutic misconception; none addressed the concept of clinician allegiance.

Among the 45 articles considered, 37 (82.2%) reported a positive effect on at least one of the studied outcomes. The efficacy of digitally supported interventions was higher for interactive multimedia interventions (13/14 articles reported a positive effect on at least one of the studied outcomes) and non-interactive multimedia interventions (8/9 articles reported a positive effect on at least one of the studied outcomes); and lower for videos (only 16/22 studies reported a positive effect). The effect was generally positive for understanding and satisfaction (75.7% and 60.0% of the studies reported a positive effect respectively), and lower for anxiety (30.8% of the studies reported a positive effect). Four out of 6 studies investigating participation reported a positive effect of the digitally-supported intervention; in two of the positive studies consent to the procedure was hypothetical. Among the 34 studies in which the researcher was present during presentation of IC, 27 (79.4%) had at least one positive outcome, compared with 10/11 (90.9%) in those in which a researcher was not present.

## Discussion

The objective of the present review was to compare the effect of digitally-supported vs non-digital IC processes on different outcomes, namely understanding, satisfaction, anxiety, participation (either real or hypothetical). Digital tools for IC published in the medical literature from January 2012 to October 2020 fell into three main categories: videos only, non-interactive multimedia tools, and interactive multimedia tools. Included studies were very heterogeneous in terms of study population, intervention, outcome measures and results. While we were unable to perform a meta-analysis due to heterogeneity in study designs, we found that the digital technologies evaluated in this review did not affect any of the outcomes negatively, and a positive—although limited—impact was observed for multimedia tools than videos only, for which impact appears lower.

We found fewer studies on digitally supported consent for research than for clinical care (surgery, therapy, vaccines, diagnostic procedures). Few articles on consent in research evaluated participation as an outcome and, in half of the cases, participation was only hypothetical. This observation suggests that studies for evaluating the impact of digital tools for the consent process, in particular for research projects, using an experimental design and including participation as an outcome should be promoted, embedding them into planned clinical trials.

Most included studies explored the added value of digital tools for obtaining consent in adult populations. Articles dedicated to consent (and assent) for studies or procedures involving children, adolescents and other minority groups (e.g. pregnant women, elderly individuals, persons with disabilities) were less represented, highlighting the need of focusing future research on these population subgroups [33, 34].

Previous reviews reported inconsistent conclusions about the use of audio-visual aids for IC [28, 31, 35]. Our review suggests that digital tools have a higher impact on IC for clinical procedures than for participation in research studies. Moreover, both in clinical research and in clinical/surgical procedures, multimedia tools seem to have a higher impact on improvement of outcomes of the IC process. One reason for this could be that the information provided in videos does not add much beyond the information already provided in person by clinicians and researchers, while combining different multimedia formats (slides, audio, video, graphics) and engaging the patient through interaction with the digital technology (mainly questions to verify understanding), seemed to improve both satisfaction and understanding (subjective and objective). The value of interaction of the patients with digital tools deserves further research, as preliminary results seem promising [36].

Presence of the researcher/clinician during the digitally-supported IC process varied across the included studies. When considering research consent, our review suggests that the presence of the researcher may enhance the efficacy of digitally supported consent processes. The mechanism for this was not established in this study, but we hypothesise that this could be due to the direct interaction between participants and researchers (e.g. question and answer). This supports the findings of Flory et al. [28], that person-to-person interaction has a high impact on understanding. On the other hand, the adoption of digital tools may facilitate addressing issues related to the IC process (e.g. therapeutic misconception, researcher bias) by guaranteeing self-standing information alongside with the presence of the researcher. Future research should focus on the role of the researcher in digitally-supported IC processes, with the aim of better specifying what is the right balance between the researcher’s contribution to participants’ comprehension of IC documents and the potential biases associated with human-mediated IC processes.

Conversely, the majority of studies on clinical and surgical procedures found that physical presence of the researcher does not add any benefit; which would lend support to the concept of a self-administered, digital consent in clinical and surgical procedures, which could reduce clinicians’ opportunity costs through time saved.

Understanding was the most described outcome, followed by satisfaction, participation and anxiety. Generally, understanding was positively affected by digitally-supported IC processes, both for research and for clinical procedures. Anxiety was not considered in any of the studies that investigated research IC, and results on the impact of digital technologies for clinical IC on anxiety were inconclusive.

Although we classified digital tools into different categories, technologies within the same category may differ in quality and/or performance. Quality could be affected by a range of factors that were usually not reported, including how the information presented was selected, the design of the tool including graphics, and the length of time given to the consent process. Outcomes and setting were also heterogeneous, making comparisons of effect between studies difficult. Different dimensions of communication should be considered when planning future studies on this topic. An attempt to standardize at least some of the outcomes would be helpful for supporting decisions to use digital tools.

We only found two studies that evaluated the effect of digital tools for research IC in developing country settings [37]. Both compared multimedia ICs (one interactive and one not) with traditional paper-based consent methods, and showed positive effect on understanding with respect to paper-based traditional ICs. In some developing country settings, patients have accepted to participate in trials despite having a limited understanding of a study, with their decision being influenced by concerns about potential consequences of refusing to participate [20]. In such contexts, it is unclear whether an improved understanding through of the digital tools would alter participation.

We also explored the inclusion of patients in the creation of the digital ICs across the included articles. Participatory approaches have previously been used to include patients in the design of IC material and processes, mainly through focus groups, in particular to address issues related to readability and understanding of the IC documents [38]. Among the studies included in our review testing digitally supported IC for research, patients were involved in the development of the IC through focus groups [39, 40], through participation in iterative review processes [41, 42] or through a direct involvement in the production of IC videos [41–43]. The use of innovative methods for a more frequent, deeper involvement of patients in the design of IC for research is advisable. We previously reported on a mixed-method approach for patient involvement, mainly based on design thinking techniques [44]. This may help to empower patients in discussing clinical decisions with clinicians and in avoiding inequities in healthcare, as suggested by other experiences in participatory healthcare [7].

This systematic review gave us some insights about the potential limitations of the adoption of digital technologies for IC. Technology evolves constantly, and the continuous change in available tools makes keeping track of tools challenging. A repository of available innovative, digital tools with a constant update system would be desirable. In addition, the digital divide has been reported to act as a barrier to access for some segments of the population such as the elderly, people from low income and minority populations, or persons with disabilities [45–47]. Additional considerations may be necessary to ensure inclusion of these populations and caution should be posed to avoid marginalization of minorities [48].

This study has a number of limitations. Study heterogeneity made inter-study comparison problematic: while we attempted to grade study quality, it was difficult to conclusively distinguish one study as being of higher quality than another, which also made it challenging to gauge the relative quality of the tools reported. We were able to broadly observe trends, but were unable to perform a meta-analysis of the results. Developing standard methods for studying and comparing digitally supported ICs (in particular for research projects) would facilitate better evaluations of innovative consent tools in the future. Moreover, we did not find a systematic evaluation of costs in any of the studies included in the review. As the investment for developing digital tools reflecting the content of the IC should be balanced with the return in terms of efficacy in improving understanding, this outcome would deserve more attention.

## Conclusions

The objective of IC is to meet patients’ needs for clear and complete information. In recent years, the use of digital tools for improving participants’/patients’ understanding and satisfaction of the IC seems to have had an impact. Digital tools, particularly interactive multimedia tools, may be useful in enabling the development of personalised IC that is tailored to an individual’s socio-cultural characteristics. Currently, studies are heterogenous. Developing standardised methods for the assessment of impact of digitally supported IC processes, including recommendations for researchers in this field, would facilitate better evaluation of innovative consent tools in the future.

## Supplementary Information

**Additional file 1: Search strategy**. The document shows the search strings used on PubMed and on EMBASE, and a list of systematic reviews screened for additional results.

**Additional file 2: Description 1**. Description of the studies included in the systematic review evaluating digitally supported IC processes for clinical research. Table showing characteristics of included studies evaluating digitally supported IC processes for clinical research and list of references.

**Additional file 3: Description 2**. Description of the studies included in the systematic review evaluating digitally supported IC processes for clinical/surgical procedures. Table showing characteristics of included studies evaluating digitally supported IC processes for clinical and surgical procedures and list of references.

## Data Availability

All data generated or analysed during this study are included in this published article.

## Abbreviations

IC: Informed consent
RCT: Randomised controlled trial
WoS: ISI web of science
PRISMA: Preferred reporting items for systematic reviews and meta-analyses
