# Supplementary material for: Digital tools in the informed consent process: a systematic review
Source: BMC Med Ethics. 2021 Feb 27;22:18. doi: 10.1186/s12910-021-00585-8 (PMC7913441; doi:10.1186/s12910-021-00585-8)
Supplement: Supplementary file 1 — Additional file 1: Search strategy. The document shows the search strings used on PubMed and on EMBASE, and a list of systematic reviews screened for additional results. [file 12910_2021_585_MOESM1_ESM.docx]

*Search string used in PubMed*

((("informed consent" OR "informed decision" OR "consent") AND ("computer" OR "multimedia" OR "multi-media" OR "tablet" OR "digital" OR "electronic" OR "video")) OR (( ("Informed Consent"[Mesh]) OR ("Informed Consent By Minors"[Mesh]) OR ("Consent Forms"[Mesh]) OR ("Third-Party Consent"[Mesh]) ) AND ( ("Computers"[MeSH]) OR ("Audiovisual Aids"[MeSH]) OR ("Smartphone"[MeSH]) OR ("Telemedicine"[MeSH]) OR ("Online Systems"[MeSH]) OR ("mobile applications"[MeSH]) OR ("multimedia"[MeSH]) OR ("internet"[MeSH]) OR ("videotape recording"[MeSH]) OR ("audiovisual aids"[MeSH]) OR ("decision making, computer assisted"[MeSH]))))

*Search string used in EMBASE*

'informed consent'/exp AND ('multimedia'/exp OR 'videorecording'/exp OR 'computer'/exp OR 'electronic device'/exp OR 'Internet'/exp OR 'audiovisual equipment'/exp OR 'mobile phone'/exp OR 'telemedicine'/exp)

*Systematic reviews screened for additional results*

Palmer BW, Lanouette NM, Jeste DV. Effectiveness of multimedia aids to enhance comprehension of research consent information: a systematic review. IRB. 2012 Nov-Dec;34(6):1-15.

Nishimura A, Carey J, Erwin PJ, Tilburt JC, Murad MH, McCormick JB. Improving understanding in the research informed consent process: a systematic review of 54 interventions tested in randomized control trials. BMC Med Ethics. 2013 Jul 23;14:28.

Nehme J, El-Khani U, Chow A, Hakky S, Ahmed AR, Purkayastha S. The use of multimedia consent programs for surgical procedures: a systematic review. Surg Innov. 2013 Feb;20(1):13-23.

Kinnersley P, Phillips K, Savage K, Kelly MJ, Farrell E, Morgan B, Whistance R, Lewis V, Mann MK, Stephens BL, Blazeby J, Elwyn G, Edwards AG. Interventions to promote informed consent for patients undergoing surgical and other invasive healthcare procedures. Cochrane Database Syst Rev. 2013 Jul 6;(7):CD009445.

Edwards AG, Naik G, Ahmed H, Elwyn GJ, Pickles T, Hood K, Playle R. Personalised risk communication for informed decision making about taking screening tests. Cochrane Database Syst Rev. 2013 Feb 28;2013(2):CD001865.

Synnot A, Ryan R, Prictor M, Fetherstonhaugh D, Parker B. Audio-visual presentation of information for informed consent for participation in clinical trials. Cochrane Database Syst Rev. 2014 May 9;2014(5):CD003717. doi: 10.1002/14651858.CD003717.pub3.

Gillies K, Cotton SC, Brehaut JC, Politi MC, Skea Z. Decision aids for people considering taking part in clinical trials. Cochrane Database Syst Rev. 2015 Nov 27;(11):CD009736.

De Sutter E, Zaçe D, Boccia S, Di Pietro ML, Geerts D, Borry P, Huys I. Implementation of Electronic Informed Consent in Biomedical Research and Stakeholders' Perspectives: Systematic Review. J Med Internet Res. 2020 Oct 8;22(10):e19129.
