# Supplementary material for: Digital tools in the informed consent process: a systematic review
Source: BMC Med Ethics. 2021 Feb 27;22:18. doi: 10.1186/s12910-021-00585-8 (PMC7913441; doi:10.1186/s12910-021-00585-8)
Supplement: Supplementary file 2 — Additional file 2: Description 1. Description of the studies included in the systematic review evaluating digitally supported IC processes for clinical research. Table showing characteristics of included studies evaluating digitally supported IC processes for clinical research and list of references. [file 12910_2021_585_MOESM2_ESM.docx]

**Description of the studies included in the systematic review evaluating digitally supported IC processes for clinical research**

| **Author** | **Year** | **Continent** | **Population** | **Intervention** | **Mock study** | **Context** |
| --- | --- | --- | --- | --- | --- | --- |
| Afolabi [1] | 2015 | Africa | adult | multimedia non interactive | No | Malaria treatment trial |
| Bergenmar [2] | 2014 | Europe | adult | multimedia non interactive | No | Oncologic clinical trial |
| Bobb [3] | 2016 | North America | adult | multimedia interactive | No | Clinical trial on prevention of pneumonia in hospitalized patients |
| Boyd [4] | 2015 | Europe | children and their parents | multimedia non interactive | No | Transgenerational prospective birth cohort |
| Brubaker [5] | 2019 | North America | adult | video | No | Clinical trial on urinary incontinence |
| Castelnuovo [6] | 2015 | Africa | adult | multimedia interactive | Yes | Generic clinical trial |
| Chapman [7] | 2020 | Australia | adult | multimedia interactive | No | Cardiovascular risk assessment study |
| Dear [8] | 2012 | Australia | adult | multimedia interactive | Yes | Oncologic clinical trial |
| Hall [9] | 2017 | North America | adult | video | Yes | HIV behavioral research study |
| Harle [10] | 2019 | North America | adult | multimedia interactive | Yes | Inclusion in a family medicine research database |
| Harmell [11] | 2012 | North America | adult | multimedia interactive | Yes | Clinical trial of an experimental cognition enhancing medication |
| Hoffner [12] | 2012 | North America | adult | video | Yes | Oncologic clinical trial |
| Jacobsen [13] | 2012 | North America | adult | video | Yes | Oncologic clinical trial |
| Jayasinghe [14] | 2019 | North America | adult | multimedia interactive | Yes | Clinical trial on falls-related anxiety |
| Jolly [15] | 2019 | Europe | adult | multimedia interactive | No | Clinical trial on people with COPD |
| Klein [16] | 2012 | North America | adult | multimedia interactive | Yes | Medical research study |
| Krishnamurti [17] | 2016 | North America | adult | video | Yes | Clinical trial on asthma treatment |
| Lindsley [18] | 2019 | North America | adult | video | No | Diabetes risk screening initiative |
| Meropol [19] | 2016 | North America | adult | multimedia interactive | Yes | Oncologic clinical trial |
| Palmer [20] | 2018 | North America | adult | video | Yes | Clinical trial for Alzheimer disease |
| Rogers [21] | 2019 | Europe | adult | video | No | Clinical trial for treatment of gout |
| Rothwell [22] | 2014 | North America | adult | video | No | Clinical trial on newborn screening |
| Rowbotham [23] | 2013 | North America | adult | multimedia interactive | No | Chemotherapy neuropathy clinical research study |
| Shelton [24] | 2015 | North America | adult | multimedia non interactive | Yes | Genomic research study |
| Simon [25] | 2016 | North America | adult | multimedia interactive | No | Recruitment in a biobank |
| Sonne [26] | 2013 | North America | adult | multimedia interactive | Yes | Generic research |
| Tait [27] | 2015 | North America | children | multimedia interactive | Yes | Generic clinical trial |
| Warriner [28] | 2016 | North America | adult | multimedia interactive | Yes | Clinical trial on osteoporosis |

1. Afolabi MO, McGrath N, D’Alessandro U, Kampmann B, Imoukhuede EB, Ravinetto RM, et al. A multimedia consent tool for research participants in the Gambia: a randomized controlled trial. Bull World Health Organ. 2015;93:320-328A.

2. Bergenmar M, Johansson H, Wilking N, Hatschek T, Brandberg Y. Audio-recorded information to patients considering participation in cancer clinical trials - a randomized study. Acta Oncol Stockh Swed. 2014;53:1197–204.

3. Bobb MR, Van Heukelom PG, Faine BA, Ahmed A, Messerly JT, Bell G, et al. Telemedicine Provides Noninferior Research Informed Consent for Remote Study Enrollment: A Randomized Controlled Trial. Acad Emerg Med Off J Soc Acad Emerg Med. 2016;23:759–65.

4. Boyd A, Tilling K, Cornish R, Davies A, Humphries K, Macleod J. Professionally designed information materials and telephone reminders improved consent response rates: evidence from an RCT nested within a cohort study. J Clin Epidemiol. 2015;68:877–87.

5. Brubaker L, Jelovsek JE, Lukacz ES, Balgobin S, Ballard A, Weidner AC, et al. Recruitment and retention: A randomized controlled trial of video-enhanced versus standard consent processes within the E-OPTIMAL study. Clin Trials Lond Engl. 2019;16:481–9.

6. Castelnuovo B, Newell K, Manabe YC, Robertson G. Multi-Media Educational Tool Increases Knowledge of Clinical Trials in Uganda. J Clin Res Bioeth. 2014;5:165.

7. Chapman N, McWhirter R, Armstrong MK, Fonseca R, Campbell JA, Nelson M, et al. Self-directed multimedia process for delivering participant informed consent. BMJ Open. 2020;10:e036977.

8. Dear RF, Barratt AL, Askie LM, Butow PN, McGeechan K, Crossing S, et al. Impact of a cancer clinical trials web site on discussions about trial participation: a cluster randomized trial. Ann Oncol Off J Eur Soc Med Oncol. 2012;23:1912–8.

9. Hall EW, Sanchez TH, Stein AD, Stephenson R, Zlotorzynska M, Sineath RC, et al. Use of Videos Improves Informed Consent Comprehension in Web-Based Surveys Among Internet-Using Men Who Have Sex With Men: A Randomized Controlled Trial. J Med Internet Res. 2017;19:e64.

10. Harle CA, Golembiewski EH, Rahmanian KP, Brumback B, Krieger JL, Goodman KW, et al. Does an interactive trust-enhanced electronic consent improve patient experiences when asked to share their health records for research? A randomized trial. J Am Med Inform Assoc JAMIA. 2019;26:620–9.

11. Harmell AL, Palmer BW, Jeste DV. Preliminary study of a web-based tool for enhancing the informed consent process in schizophrenia research. Schizophr Res. 2012;141:247–50.

12. Hoffner B, Bauer-Wu S, Hitchcock-Bryan S, Powell M, Wolanski A, Joffe S. “Entering a Clinical Trial: Is it Right for You?”: a randomized study of The Clinical Trials Video and its impact on the informed consent process. Cancer. 2012;118:1877–83.

13. Jacobsen PB, Wells KJ, Meade CD, Quinn GP, Lee J-H, Fulp WJ, et al. Effects of a brief multimedia psychoeducational intervention on the attitudes and interest of patients with cancer regarding clinical trial participation: a multicenter randomized controlled trial. J Clin Oncol Off J Am Soc Clin Oncol. 2012;30:2516–21.

14. Jayasinghe N, Moallem BI, Kakoullis M, Ojie M-J, Sar-Graycar L, Wyka K, et al. Establishing the Feasibility of a Tablet-Based Consent Process with Older Adults: A Mixed-Methods Study. The Gerontologist. 2019;59:124–34.

15. Jolly K, Sidhu M, Bower P, Madurasinghe V. Improving recruitment to a study of telehealth management for COPD: a cluster randomised controlled “study within a trial” (SWAT) of a multimedia information resource. Trials. 2019;20:453.

16. Klein DW, Schartz HA. Instructional strategies to improve informed consent in healthcare research: Pilot study of interactivity and multimedia. In: American Educational Research Association National Conference, Vancouver, British Columbia, Canada. 2012.

17. Krishnamurti T, Argo N. A Patient-Centered Approach to Informed Consent: Results from a Survey and Randomized Trial. Med Decis Mak Int J Soc Med Decis Mak. 2016;36:726–40.

18. Lindsley KA. Improving quality of the informed consent process: Developing an easy-to-read, multimodal, patient-centered format in a real-world setting. Patient Educ Couns. 2019;102:944–51.

19. Meropol NJ, Wong Y-N, Albrecht T, Manne S, Miller SM, Flamm AL, et al. Randomized Trial of a Web-Based Intervention to Address Barriers to Clinical Trials. J Clin Oncol Off J Am Soc Clin Oncol. 2016;34:469–78.

20. Palmer BW, Harmell AL, Dunn LB, Kim SY, Pinto LL, Golshan S, et al. Multimedia Aided Consent for Alzheimer’s Disease Research. Clin Gerontol. 2018;41:20–32.

21. Rogers A, Flynn RWV, Mackenzie IS, MacDonald TM. Does the provision of a DVD-based audio-visual presentation improve recruitment in a clinical trial? A randomised trial of DVD trial invitations. BMC Med Res Methodol. 2019;19:24.

22. Rothwell E, Wong B, Rose NC, Anderson R, Fedor B, Stark LA, et al. A randomized controlled trial of an electronic informed consent process. J Empir Res Hum Res Ethics JERHRE. 2014;9:1–7.

23. Rowbotham MC, Astin J, Greene K, Cummings SR. Interactive informed consent: randomized comparison with paper consents. PloS One. 2013;8:e58603.

24. Shelton AK, Freeman BD, Fish AF, Bachman JA, Richardson LI. A computer-based education intervention to enhance surrogates’ informed consent for genomics research. Am J Crit Care Off Publ Am Assoc Crit-Care Nurses. 2015;24:148–55.

25. Simon CM, Klein DW, Schartz HA. Interactive multimedia consent for biobanking: a randomized trial. Genet Med Off J Am Coll Med Genet. 2016;18:57–64.

26. Sonne SC, Andrews JO, Gentilin SM, Oppenheimer S, Obeid J, Brady K, et al. Development and pilot testing of a video-assisted informed consent process. Contemp Clin Trials. 2013;36:25–31.

27. Tait AR, Voepel-Lewis T, Levine R. Using digital multimedia to improve parents’ and children’s understanding of clinical trials. Arch Dis Child. 2015;100:589–93.

28. Warriner AH, Foster PJ, Mudano A, Wright NC, Melton ME, Sattui SE, et al. A pragmatic randomized trial comparing tablet computer informed consent to traditional paper-based methods for an osteoporosis study. Contemp Clin Trials Commun. 2016;3:32–8.
