# Supplementary material for: Digital tools in the informed consent process: a systematic review
Source: BMC Med Ethics. 2021 Feb 27;22:18. doi: 10.1186/s12910-021-00585-8 (PMC7913441; doi:10.1186/s12910-021-00585-8)
Supplement: Supplementary file 3 — Additional file 3: Description 2. Description of the studies included in the systematic review evaluating digitally supported IC processes for clinical/surgical procedures. Table showing characteristics of included studies evaluating digitally supported IC processes for clinical and surgical procedures and list of references. [file 12910_2021_585_MOESM3_ESM.docx]

**Description of the studies included in the systematic review evaluating digitally supported IC processes for clinical/surgical procedures**

| **Author** | **Year** | **Continent** | **Population** | **Intervention** | **Mock study** | **Context** |
| --- | --- | --- | --- | --- | --- | --- |
| Al-Silwadi [1] | 2015 | Europe | adult | video | No | Orthodontic treatment |
| Baenninger [2] | 2018 | Europe | adult | video | No | Refractive laser treatment |
| Bethune [3] | 2018 | North America | adult | multimedia interactive | No | Lumbar spine or cervical spine decompression for degenerative disease, craniotomy for brain tumour or trigeminal neuralgia treatment |
| Beulen [4] | 2016 | Europe | adult | multimedia interactive | No | Prenatal testing for fetal chromosomal abnormalities |
| Bishop [5] | 2019 | Europe | adult | multimedia interactive | Yes | Acupuncture for back pain |
| Book [6] | 2020 | Europe | adult | multimedia non interactive | No | Inguinal hernia surgery in children |
| Bowers [7] | 2017 | North America | adults | video | No | Peripherally inserted central venous catheter, Hickman catheter insertion, peripheral angioplasty with or without stenting, or endovascular aneurysm repair |
| Brandel [8] | 2017 | North America | adult | multimedia interactive | No | Breast reconstruction, breast reduction, or abdominoplasty |
| Delcambre [9] | 2019 | North America | adult | video | No | Mohs micrographic surgery for diagnoses of basal cell carcinoma, squamous cell carcinoma, and/or melanoma in situ |
| Dunbar [10] | 2019 | North America | adult | video | No | Lumbar puncture in children |
| Ellett [11] | 2014 | Oceania | adult | multimedia non interactive | No | Operative gynecologic laparoscopy |
| Fraval [12] | 2015 | Oceania | adult | multimedia interactive | No | Knee arthroplasty, total hip arthroplasty, knee arth-  roscopy, shoulder arthroscopy and ACL reconstruction |
| Gordon [13] | 2017 | North America | adult | multimedia interactive | Yes | Increased risk donors for kidney transplant |
| Günay [14] | 2015 | Asia | adult | multimedia non interactive | No | Bornchoscopy |
| Ham [15] | 2016 | Asia | adult | multimedia non interactive | No | Green-light high-performance system  photoselective vaporization of the prostate |
| Huber [16] | 2013 | Europe | adult | multimedia interactive | No | Radical prostatectomy |
| Lattuca [17] | 2018 | Europe | adult | video | No | Coronary angiography |
| Li [18] | 2020 | Asia | adult | video | No | Peripherally inserted  central catheters |
| Lin [19] | 2018 | Asia | adult | video | No | Trauma-related debridement surgery |
| Mednick [20] | 2016 | North America | adult | video | No | Intravenous fluorescein angiography |
| Ohman [21] | 2012 | Europe | adult | video | Yes | Pregnancy second trimester ultrasound |
| Pallett [22] | 2018 | North America | adult | video | No | Hysterectomy |
| Pawlak [23] | 2015 | North America | adolescent | multimedia non interactive | No | Orthodontic treatment |
| Pot [24] | 2017 | Europe | adult | multimedia non interactive | Yes | HPV vaccination |
| Prochazka [25] | 2014 | North America | adult | multimedia non interactive | No | Total hip arthroplasty, carotid endarterectomy, laparoscopic cholecystectomy, and radical prostatectomy |
| Roberts [26] | 2016 | North America | adult | multimedia interactive | Yes | Thyroidectomy |
| Saglam [27] | 2020 | Asia | adult | video | No | Bariatric surgery |
| Sariturk [28] | 2017 | Asia | adolescent/adult | video | No | Stem cell receiving and donation |
| Schauer [29] | 2019 | Australia | adult | video | No | Gastroscopy or colonoscopy |
| Schlechtweg [30] | 2014 | Europe | adult | multimedia interactive | No | Routine MRI |
| Shukla [31] | 2012 | North America | adult | video | No | Cataract surgery |
| Siu [32] | 2016 | North America | adult | multimedia interactive | No | Endoscopic sinus surgery |
| Spencer [33] | 2015 | North America | children | multimedia non interactive | No | Ketamine sedation for fracture reduction of pediatric patients |
| Sugand [34] | 2019 | Europe | adults | multimedia interactive | No | Knee or shoulder surgery |
| Tait [35] | 2014 | North America | adult | multimedia interactive | No | Cardiac catheterization |
| Thornton [36] | 2012 | North America | children/adults | video | No | Organ donation |
| Tipotsch-Maca [37] | 2016 | Europe | adult | video | No | Cataract surgery |
| Truong [38] | 2020 | Oceania | adult | multimedia interactive | No | Elective caesarean section |
| Vo [39] | 2018 | North America | adult | video | No | Cataract surgery |
| Winter [40] | 2016 | Oceania | adult | video | No | Cystoscopy and stent insertion |
| Wollinger [41] | 2012 | Europe | adult | multimedia interactive | No | Cataract surgery |
| Xia [42] | 2019 | Asia | adult | video | No | Endoscopic retrograde cholangiopancreatography |
| Yeh [43] | 2017 | North America | children/their parents | video | No | Gastrointestinal endoscopy |
| Yin [44] | 2015 | North America | adult | multimedia non interactive | No | Knee arthroscopy |
| Zhang [45] | 2019 | North America | adult | video | No | Cataract surgery |

1. Al-Silwadi FM, Gill DS, Petrie A, Cunningham SJ. Effect of social media in improving knowledge among patients having fixed appliance orthodontic treatment: A single-center randomized controlled trial. Am J Orthod Dentofac Orthop Off Publ Am Assoc Orthod Its Const Soc Am Board Orthod. 2015;148:231–7.

2. Baenninger PB, Faes L, Kaufmann C, Reichmuth V, Bachmann LM, Thiel MA. Efficiency of video-presented information about excimer laser treatment on ametropic patients’ knowledge and satisfaction with the informed consent process. J Cataract Refract Surg. 2018;44:1426–30.

3. Bethune A, Davila-Foyo M, Valli M, da Costa L. e-Consent: approaching surgical consent with mobile technology. Can J Surg J Can Chir. 2018;61:339–44.

4. Beulen L, van den Berg M, Faas BH, Feenstra I, Hageman M, van Vugt JM, et al. The effect of a decision aid on informed decision-making in the era of non-invasive prenatal testing: a randomised controlled trial. Eur J Hum Genet EJHG. 2016;24:1409–16.

5. Bishop FL, Greville-Harris M, Bostock J, Din A, Graham CA, Lewith G, et al. Informing Adults With Back Pain About Placebo Effects: Randomized Controlled Evaluation of a New Website With Potential to Improve Informed Consent in Clinical Research. J Med Internet Res. 2019;21:e9955.

6. Book F, Goedeke J, Poplawski A, Muensterer OJ. Access to an online video enhances the consent process, increases knowledge, and decreases anxiety of caregivers with children scheduled for inguinal hernia repair: A randomized controlled study. J Pediatr Surg. 2020;55:18–28.

7. Bowers N, Eisenberg E, Montbriand J, Jaskolka J, Roche-Nagle G. Using a multimedia presentation to improve patient understanding and satisfaction with informed consent for minimally invasive vascular procedures. Surg J R Coll Surg Edinb Irel. 2017;15:7–11.

8. Brandel MG, Reid CM, Parmeshwar N, Dobke MK, Gosman AA. Efficacy of a Procedure-Specific Education Module on Informed Consent in Plastic Surgery. Ann Plast Surg. 2017;78 5 Suppl 4:S225–8.

9. Delcambre M, Haynes D, Hajar T, Golden S, Bar A, Latour E, et al. Using a Multimedia Tool for Informed Consent in Mohs Surgery: A Randomized Trial Measuring Effects on Patient Anxiety, Knowledge, and Satisfaction. United States; 2020.

10. Dunbar M, Paton G, Singhal A. An Educational Video Improves Consent in Pediatric Lumbar Puncture: A Randomized Control Trial. Pediatr Neurol. 2019;100:74–9.

11. Ellett L, Villegas R, Beischer A, Ong N, Maher P. Use of a multimedia module to aid the informed consent process in patients undergoing gynecologic laparoscopy for pelvic pain: randomized controlled trial. J Minim Invasive Gynecol. 2014;21:602–11.

12. Fraval A, Chandrananth J, Chong YM, Coventry LS, Tran P. Internet based patient education improves informed consent for elective orthopaedic surgery: a randomized controlled trial. BMC Musculoskelet Disord. 2015;16:14.

13. Gordon EJ, Sohn M-W, Chang C-H, McNatt G, Vera K, Beauvais N, et al. Effect of a Mobile Web App on Kidney Transplant Candidates’ Knowledge About Increased Risk Donor Kidneys: A Randomized Controlled Trial. Transplantation. 2017;101:1167–76.

14. Günay E, Baki ED, Kokulu S, Ulaşlı SS, Öz G, Akar O, et al. Impact of multimedia information on bronchoscopy procedure: is it really helpful? Ann Thorac Med. 2015;10:34–7.

15. Ham DY, Choi WS, Song SH, Ahn Y-J, Park HK, Kim HG, et al. Prospective Randomized Controlled Study on the Efficacy of Multimedia Informed Consent for Patients Scheduled to Undergo Green-Light High-Performance System Photoselective Vaporization of the Prostate. World J Mens Health. 2016;34:47–55.

16. Huber J, Ihrig A, Yass M, Bruckner T, Peters T, Huber CG, et al. Multimedia support for improving preoperative patient education: a randomized controlled trial using the example of radical prostatectomy. Ann Surg Oncol. 2013;20:15–23.

17. Lattuca B, Barber-Chamoux N, Alos B, Sfaxi A, Mulliez A, Miton N, et al. Impact of video on the understanding and satisfaction of patients receiving informed consent before elective inpatient coronary angiography: A randomized trial. Am Heart J. 2018;200:67–74.

18. Li J, Huang X-F, Luo J-L, Zhang J-Y, Liang X-L, Huang C-L, et al. Effect of video-assisted education on informed consent and patient education for peripherally inserted central catheters: a randomized controlled trial. J Int Med Res. 2020;48:300060520947915.

19. Lin Y-K, Chen C-W, Lee W-C, Cheng Y-C, Lin T-Y, Lin C-J, et al. Educational video-assisted versus conventional informed consent for trauma-related debridement surgery: a parallel group randomized controlled trial. BMC Med Ethics. 2018;19:23.

20. Mednick Z, Irrcher I, Hopman WM, Sharma S. Assessing a narrated white board animation as part of the consent process for intravenous fluorescein angiography: a randomized educational study. Can J Ophthalmol J Can Ophtalmol. 2016;51:471–5.

21. Ohman SG, Björklund U, Marsk A. Does an informational film increase women’s possibility to make an informed choice about second trimester ultrasound? Prenat Diagn. 2012;32:833–9.

22. Pallett AC, Nguyen BT, Klein NM, Phippen N, Miller CR, Barnett JC. A randomized controlled trial to determine whether a video presentation improves informed consent for hysterectomy. Am J Obstet Gynecol. 2018;219:277.e1-277.e7.

23. Pawlak CE, Fields HWJ, Beck FM, Firestone AR. Orthodontic informed consent considering information load and serial position effect. Am J Orthod Dentofac Orthop Off Publ Am Assoc Orthod Its Const Soc Am Board Orthod. 2015;147:363–72.

24. Pot M, Paulussen TG, Ruiter RA, Eekhout I, de Melker HE, Spoelstra ME, et al. Effectiveness of a Web-Based Tailored Intervention With Virtual Assistants Promoting the Acceptability of HPV Vaccination Among Mothers of Invited Girls: Randomized Controlled Trial. J Med Internet Res. 2017;19:e312.

25. Prochazka AV, Fink AS, Bartenfeld D, Henderson WG, Nyirenda C, Webb A, et al. Patient perceptions of surgical informed consent: is repeat back helpful or harmful? J Patient Saf. 2014;10:140–5.

26. Roberts KJ, Revenson TA, Urken ML, Fleszar S, Cipollina R, Rowe ME, et al. Testing with feedback improves recall of information in informed consent: A proof of concept study. Patient Educ Couns. 2016;99:1377–81.

27. Saglam K, Kayaalp C, Aktas A, Sumer F. Educational Video Addition to the Bariatric Surgery Informed Consent Process: a Randomized Controlled Trial. Obes Surg. 2020;30:2693–9.

28. Sarıtürk Ç, Gereklioğlu Ç, Korur A, Asma S, Yeral M, Solmaz S, et al. Effectiveness of Visual Methods in Information Procedures for Stem Cell Recipients and Donors. Turk J Haematol Off J Turk Soc Haematol. 2017;34:321–7.

29. Schauer C, Floyd T, Chin J, Vandal A, Lampen-Smith A. Video or verbal? A randomised trial of the informed consent process prior to endoscopy. N Z Med J. 2019;132:57–68.

30. Schlechtweg PM, Hammon M, Giese D, Heberlein C, Uder M, Schwab SA. iPad-based patient briefing for radiological examinations-a clinical trial. J Digit Imaging. 2014;27:479–85.

31. Shukla AN, Daly MK, Legutko P. Informed consent for cataract surgery: patient understanding of verbal, written, and videotaped information. J Cataract Refract Surg. 2012;38:80–4.

32. Siu JM, Rotenberg BW, Franklin JH, Sowerby LJ. Multimedia in the informed consent process for endoscopic sinus surgery: A randomized control trial. The Laryngoscope. 2016;126:1273–8.

33. Spencer SP, Stoner MJ, Kelleher K, Cohen DM. Using a Multimedia Presentation to Enhance Informed Consent in a Pediatric Emergency Department. Pediatr Emerg Care. 2015;31:572–6.

34. Sugand K, Malik HH, Newman S, Spicer D, Reilly P, Gupte CM. Does using anatomical models improve patient satisfaction in orthopaedic consenting? Single-blinded randomised controlled trial. Surg J R Coll Surg Edinb Irel. 2019;17:146–55.

35. Tait AR, Voepel-Lewis T, Chetcuti SJ, Brennan-Martinez C, Levine R. Enhancing patient understanding of medical procedures: evaluation of an interactive multimedia program with in-line exercises. Int J Med Inf. 2014;83:376–84.

36. Thornton JD, Alejandro-Rodriguez M, León JB, Albert JM, Baldeon EL, De Jesus LM, et al. Effect of an iPod video intervention on consent to donate organs: a randomized trial. Ann Intern Med. 2012;156:483–90.

37. Tipotsch-Maca SM, Varsits RM, Ginzel C, Vecsei-Marlovits PV. Effect of a multimedia-assisted informed consent procedure on the information gain, satisfaction, and anxiety of cataract surgery patients. J Cataract Refract Surg. 2016;42:110–6.

38. Truong A, Ellett L, Hicks L, Pell G, Walker SP. Multimedia in improving informed consent for caesarean section: A randomised controlled trial. Aust N Z J Obstet Gynaecol. 2020;60:683–9.

39. Vo TA, Ngai P, Tao JP. A randomized trial of multimedia-facilitated informed consent for cataract surgery. Clin Ophthalmol Auckl NZ. 2018;12:1427–32.

40. Winter M, Kam J, Nalavenkata S, Hardy E, Handmer M, Ainsworth H, et al. The use of portable video media vs standard verbal communication in the urological consent process: a multicentre, randomised controlled, crossover trial. BJU Int. 2016;118:823–8.

41. Wollinger C, Hirnschall N, Findl O. Computer-based tutorial to enhance the quality and efficiency of the informed-consent process for cataract surgery. J Cataract Refract Surg. 2012;38:655–9.

42. Xia T, Zhu YB, Zeng YB, Chen C, Wang SL, Zhao SB, et al. Video education can improve awareness of risks for patients undergoing endoscopic retrograde cholangiopancreatography: A randomized trial. J Dig Dis. 2019;20:656–62.

43. Yeh DM, Chun S, Terrones L, Huang JS. Using media to improve the informed consent process for youth undergoing pediatric endoscopy and their parents. Endosc Int Open. 2017;5:E41–6.

44. Yin B, Goldsmith L, Gambardella R. Web-Based Education Prior to Knee Arthroscopy Enhances Informed Consent and Patient Knowledge Recall: A Prospective, Randomized Controlled Study. J Bone Joint Surg Am. 2015;97:964–71.

45. Zhang MH, Haq ZU, Braithwaite EM, Simon NC, Riaz KM. A randomized, controlled trial of video supplementation on the cataract surgery informed consent process. Germany; 2019.
